# Spoofing Attacks on FMCW Radars with Low-Cost Backscatter Tags

**DOI:** 10.3390/s22062145

**Published:** 2022-03-10

**Authors:** Antonio Lazaro, Arnau Porcel, Marc Lazaro, Ramon Villarino, David Girbau

**Affiliations:** Department of Electronics, Electrics and Automatic Control Engineering, Rovira i Virgili University, 43007 Tarragona, Spain; arnau.porcel@fundacio.urv.cat (A.P.); marc.lazaro@urv.cat (M.L.); ramon.villarino@urv.cat (R.V.); david.girbau@urv.cat (D.G.)

**Keywords:** automotive radar, mm wave radar, FMCW, spoofing, interference, backscatter

## Abstract

This work studies the feasibility of using backscatter-modulated tags to introduce false information into a signal received by a frequency-modulated continuous-wave (FMCW) radar. A proof-of-concept spoofing device was designed in the 24 GHz ISM band. The spoofing device was based on an amplifier connected between two antennas, and modulation was carried out by switching the amplifier bias. The use of an amplifier allowed us to increase the level of spoofing signal compared with other modulated backscattering methods. The simulated and experimental results show that our method has the ability to generate a pair of false targets at different ranges and velocities depending on the modulation frequency of the chosen tag, since sidebands appear due to this modulation. Countermeasures to detect the spoofing attack based on changes in the slope of the frequency sweep between frames are also proposed.

## 1. Introduction

### 1.1. Background and Motivation

Frequency-modulated continuous-wave (FMCW) radars are widely used in short-range applications as an alternative to pulsed radars. Compared with continuous-wave (CW) radars, these radars allow a user to also determine the distance. The range of applications for FMCW radars has increased significantly in recent years to different fields. Some of these applications include altimeters for unmanned aerial vehicles (UAV) [1] and aircrafts, drone detection, traffic radars, remote monitoring of human vital signs [2], accurate distance measurements [3], human activity classification [4], gesture recognition [5], and in-vehicle occupancy sensors [6]. However, one of the most significant applications is its use in the automotive field.

Automotive radars include technologies that are part of modern advanced driver assistance systems (ADAS), camera-based computer vision systems, or, recently, LiDARS. Radars have some advantages, such as the signal not being affected by bad weather conditions (e.g., rain or fog) or dust, especially in computer vision systems or LiDARS [7]. Therefore, these systems are complementary, and automotive radars are considered a key technology in autonomous driving as they detect surrounding obstacles. Automotive radars available on the market operate in the millimeter-wave (mm wave) band. Short-range radars operate at the 24 GHz band, although lately, they are also moving to the 79–81 GHz band. Medium- and long-range radars operate at 77–81 GHz and 76–77 GHz, respectively. Most automotive radars are based on frequency-modulated continuous-wave (FMCW) radars that are capable of measuring the range and velocity of targets. Moreover, modern automotive radars use beam-forming and MIMO techniques [8] to detect the angle of arrival, thus allowing for an improvement in the positioning of objects. The use of the mm wave band offers the possibility of reaching a high bandwidth, which translates into a higher resolution range. As a consequence of the development of millimeter-wave band technology for automotive radars and new mobile generations, the cost of these radars has been significantly reduced and their use has been extended to other industrial applications using alternative frequency bands such as the 24 GHz ISM band. Another consequence of operating in the mm wave region of the spectrum is that automotive radars are currently not significantly affected by interference from other systems. These radars use a part of the spectrum that other communication systems do not use. This technology will become more widespread in the future, and both interference and attacks will become a major security concern that should be addressed. Automotive radars are safety elements in vehicles. Therefore, any cyberattack on these systems can pose a significant risk to safety. These concerns will become more important when automatic vehicles become commercially available.

These cyberattacks intentionally target the malfunctioning of radars. They can be classified into three main types [9]. The first type is radio jamming, which is the intentional transmission of radio frequency signals to saturate or block the receiver by decreasing the signal-to-noise ratio [10]. A transmitter, tuned to the same frequency band, sends signals with enough power to the radar. The most common types of jamming signals are continuous-wave (CW) signals, random noise, or modulated signals [11,12]. The purpose of jamming in communication systems is to inhibit the reception of signals. Radar jamming has been extensively studied in the literature [13,14] and is usually linked to electronic warfare (EW) [15], which has recently attracted interest in blocking transmissions from drones that invade restricted areas such as airports [16,17,18,19]. Jamming attacks on an automotive radar are described in [20].

**Figure 1:**
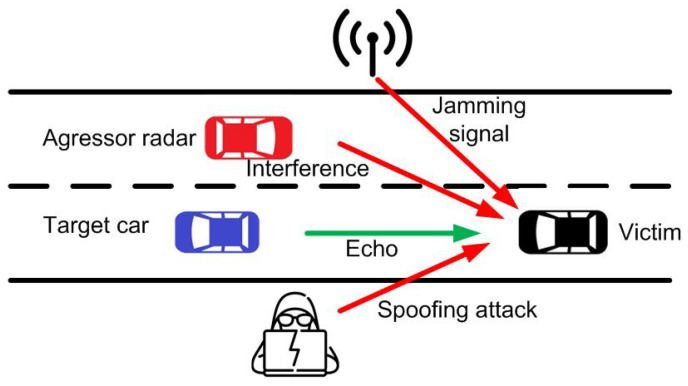
Illustration of a road scenario where the victim’s radar receives the echo signal from a target and is interfered with by another radar, a jamming transmitter, and an spoofing attack.

The second type of attack is interferences, which are the introduction of unwanted signals into the radar band from other systems. The difference from radio jamming is that interferences are unintentional, and, in this case, the interference comes from communication signals of other users, e.g., from other systems sharing the same frequency band. Some differences in the automotive field can be seen when compared with other radar systems (such as military or aeronautic radars) due to the specificity of vehicular networks [21]. The interference of other vehicles equipped with a radar that works at the same frequency band [22] (see Figure 1) is a potential source of interference that has been addressed in the literature [23,24,25]. A solution that avoids this type of interference is the use of different modulation strategies that make the interference uncorrelated with the radar signal itself [26], or beamforming methods [27,28].

**Figure 2:**
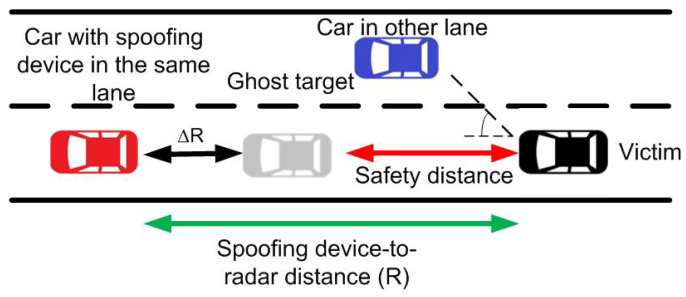
Scheme of a car-following model where the victim radar experiences a spoofing attack from a device installed in a car in the same lane, introducing a false target less than a safe distance away.

Finally, the third type of attack is known as spoofing, which is the retransmission of radar signals to provide false information to the radar, misleading it in target detection, and increasing the risk of collision [29]. Figure 1 illustrates a typical road scenario showing the three types of attacks on a victim radar that can confuse the detection of the real target. Since automotive radars have a direction-of-arrival detection capability, for a spoofing attack to be damaging, the spoofing device should be placed in the same lane as the victim radar. For example, Figure 2 shows a spoofing attack on the car-following model, where the victim radar is located behind the attacker’s vehicle. The attacker has the ability to generate an artificial target less than a safe distance away, perform the attack, and cause an alarm in the victim’s anti-collision system. A spoofing attack from a different lane is detectable due to the wider angle of arrival.

In this work, jamming attacks are not considered since modern radars are designed to minimize their impact. In addition, jamming is relatively easy to detect. Thus, this work is focused on attacks caused by spoofing.

### 1.2. Related Work

Studies on cyberattacks on FMCW radars, especially in the automotive field, have been recently reported. In [30,31,32], the methods for detecting and mitigating spoofing attacks against an automotive radar based on simulations of the car-following model have been proposed. However, few experimental examples of spoofing attacks can be found in the literature. Most of them are based on simulated platforms using software-defined radio (SDR) and evaluation radar kits used as spoofing devices. Spoofing attacks against vehicular FMCW radar have recently been studied in [33], where experimental results have been implemented using SDR at 6 GHz. Experimental studies of cyberattacks introducing an interference from another source (a radar kit at 77 GHz from Texas Instruments) and strategies based on the design of a detection threshold to minimize the attacks have also recently been presented in [34]. A spoofing device based on the use of another radar that retransmits a replica of the signal has been presented in [35] for distance spoofing. A method for interference and spoofing mitigation based on a random-frequency hopping radar (BlueFMCW) has recently been proposed in [36], including experimental validation with a 77 GHz radar kit. In [37], a spoofing device prototype based on a phase-quadrature (IQ) mixer for FMCW radar at 5.8 GHz has been presented. The local oscillator was extracted from the amplified radar signal and was mixed with a low-frequency spoofing signal. The signal at the output of the mixer was amplified and transmitted through the antenna.

Spoofing attacks can also be performed on other radar applications [38,39,40]. In particular, there is a growing interest in detecting unmanned aerial vehicles (UAV) [41] and one of the detection techniques proposed is the use of FMCW radars, which are susceptible to suffer attacks [42,43,44].

Backscattering communication is a data-transmission method based on the modulation of an incident RF signal from a reader. Backscattering is extensively used in radio frequency identification systems (RFID) and in low-power ambient backscatter, also known as RF backscatter. Backscatter communication has proven to be indispensable for the deployment of Internet of Things (IoT). As communications technology in the millimeter-wave frequency band has matured, the interest in mm wave ID (MMID) and in communications applications using backscatters at these bands has grown in recent years [45,46,47]. These systems are characterized by the use of small directional reader antennas and high bandwidth for data handling. Radars with small modifications can be used as readers in these backscattering systems [48,49,50,51]. Therefore, backscatters can be used for distance measurements, localization, or sensing in combination with FMCW and UWB radars.

### 1.3. Contribution

The objective of this paper is to study a hypothetical spoofing attack on an FMCW radar using low-cost devices. The main contribution of this work is based on a novel and simple concept, consisting of using a semi-passive modulated transponder or tag, for spoofing applications, instead of using SDR or other radars to generate spoofing signals, as presented in other works found in the literature. Simulated and experimental results show that by changing the modulation frequency of the transponder, it is possible to generate ghost targets at different distances and velocities in order to confuse the radar. Transponder modulation can be easily implemented with a low-cost microcontroller without the need to implement complex modulations or complicated algorithms. Compared to passive backscatters on reflection, the use of a gain-modulated transponder allows the level of the spoofing signal to be increased near to the echo of a small vehicle. Therefore, the spoofing signal can exceed the threshold level set in the radars to eliminate clutter.

A proof-of-concept device has been designed using commercial off-the-shelf (COST) components and 3D-printed directive lens antennas integrated into a case. In addition, the device is portable and has low power consumption due the use of low-power SiGe monolithic microwave integrated circuit (MMIC) amplifiers (<12 mA at 3 V). Experimental results of false targets generation and detection are demonstrated using a 24 GHz FMCW radar. The proposed spoofing device does not require a local oscillator; therefore, it can be scaled to other radar bands such as those licensed for automotive radar at 77 GHz. Therefore, the proposed device can be useful in experimentally testing countermeasure algorithms to protect these radars from spoofing attacks.

The paper is organized as follows. Section 2 describes the detection of modulated backscatter using an FMCW radar. A relatively simple and inexpensive device used to confuse the radar is presented. Section 3 describes the design of the proposed solution. Simulated and experimental results are shown in Section 4. Some countermeasures used to detect the presence of a spoofing device based on backscatters are proposed in Section 5. Finally, conclusions are provided in Section 6.

## 2. System Overview and Theoretical Background

### FMCW Radar and Backscatter Detection

**Figure 3:**
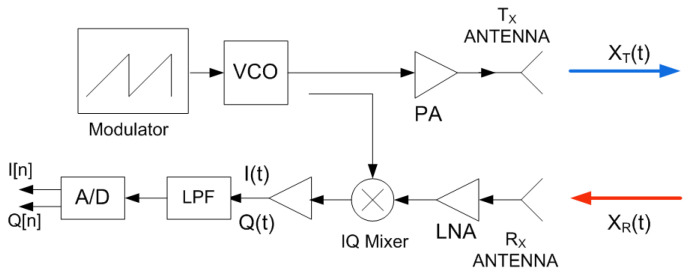
Block diagram of a generic FMCW radar used in the analysis.

FMCW radars, such as those used in the automotive field, are characterized by the ability to measure range and velocity. By adding MIMO structures based on virtual arrays [52], modern automotive radars can estimate the direction of arrival (DOA). This work focuses on a basic one-channel FMCW radar. Figure 3 shows the main blocks of an FMCW radar. The radar transmitter basically consists of a VCO in which the control input is modulated by a modulator. Today, these modulators are implemented with fast digital-to-analogue converters (DAC) that allow different waveforms to be configured. The most commonly used waveform is a single slope sawtooth. As a result, the transmitted signal is a chirp-type in which the frequency varies from a minimum ($f_{min}$) to a maximum ($f_{max}$). The bandwidth ($B=f_{max}-f_{min}$) determines the range resolution, ΔR=c/(2B), with *c* being the speed of light in a vacuum. Therefore, the range resolution of the highest automotive bands (77–81 GHz) is better due to the higher available bandwidth. Another important parameter is the sweep slope defined as μ=B/T, with *T* being the sweep time. The transmitted wave propagates to the target and is backscattered and received by the radar receiver. The FMCW radar front-end consists of a down-converter phase-quadrature mixer, the IF amplifiers, and the antialiasing low-pass filters. The phase and quadrature signals are sampled at a rate $f_{s}$ using fast analogue-to-digital converters (ADCs). The samples from the phase I[n] and quadrature Q[n] channels are sent to the processor (microcontroller or DSP), which performs the signal processing and target detection. The methods used to combat interference between radars are based on the variations in the modulation waveform. Thus, the receiver is able to distinguish the signals sent by the radar itself, since it uses parameters for which interference from other radars can be detected [23,26].

In [33,35,38], spoofing devices based on a phase-quadrature modulator with an oscillator were proposed. They proposed sending artificial chirp signals to generate ghost responses with the same parameters as the victim radar but without transmitting the signals. Therefore, detecting the IF signal to synchronize and tune the attack parameters is necessary. The idea is to use another radar device with the same parameter as the victim’s radar. These spoofing devices are designed to generate errors in range. As a countermeasure against this distance-spoofing attack, a random-chirp modulation was proposed to make the modulation parameters difficult to tune.

This work investigates a possible attack using backscatter devices. The backscatter returns the radar signal but introduces a modulation that generates a false response. The retransmission of the same signal is independent of the type of radar and modulation used by it, which makes applying countermeasures difficult. In addition, the devices are simpler than those proposed in previous work and are based on low-cost components.

**Figure 4:**
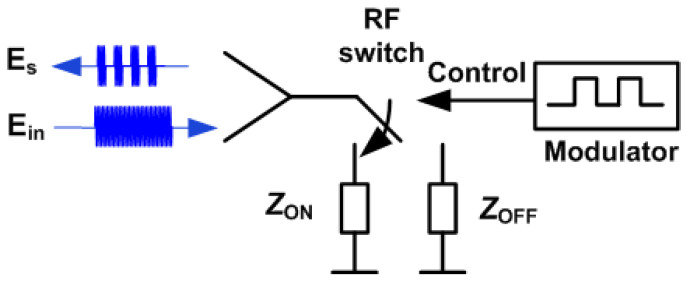
Block diagram of a passive modulated backscatter in reflection based on a microwave switch.

Figure 4 shows a schema of a simple backscatter typically used in RFID tags or in communication systems based on backscattering. The communication principle in these systems is based on load modulation, which consists of switching the antenna load between two impedances ($Z_{\mathrm{ON}}$ and $Z_{\mathrm{OFF}}$) for ON and OFF switch states, respectively, that have high reflection coefficients (e.g., short-circuit and open-circuit). A low-frequency modulator is responsible for controlling the switch transmitting the data to the reader. The result is the change in the backscattered field and therefore the modulation of the radar cross section of the tag. The modulation is carried out with an RF switch that works similar to a mixer. At microwave and millimeter-wave bands, this switch can be implemented using PIN or Schottky diodes. For example, Refs. [48,49] describe modulated backscatters for an FMCW radar-based range using reflection backscatters based on diodes.

From the antenna scattering theory, the backscattered field can be expressed as the sum of two main contributions: the structural mode ${\bar{E}}_{est}$, which depends on the shape and materials that support the tag as a passive device, and the antenna or tag mode ${\bar{E}}_{m}$, which is the field re-radiated by the antenna that depends on the modulation. Therefore, the backscattered field ${\bar{E}}_{s}\left(Z_{L}\right)$ can be written as a function of the antenna load $Z_{L}$ [53,54]:

(1) $${\bar{E}}_{s}\left(Z_{L}\right)=Re\left[\left({\bar{E}}_{est}+{\bar{E}}_{m}\cdot \Gamma_{L}\right)e^{j2\pi f_{c}t}\right]$$

where Re() denotes the real part operator, $f_{c}$ is the carrier frequency of the incoming field, and $\Gamma_{L}$ is the power reflection coefficient that can be obtained from the load and the antenna impedance $Z_{a}$ using the following [55]:

(2) $$\Gamma_{L}=\frac{Z_{L}-Z_{a}^{*}}{Z_{L}+Z_{a}}$$

The antenna mode is composed of multiple sidebands located at the harmonics of the modulation frequency (switching rate of the two impedances). The amplitude of these sidebands depends on the Fourier coefficients $c_{n}$ (e.g., square wave coefficients). The power reflection coefficient can be written as a Fourier series because it is a periodic function of the modulation frequency [50]:

(3) $$\Gamma_{L}=\Delta \Gamma \cdot \sum_{k=0}^{\infty }\prod \left(\frac{t-kT_{m}}{T_{m}}\right)=\sum_{n=-\infty }^{\infty }c_{n}e^{\frac{j2\pi n}{T_{m}}}$$

where ∏(t) is the rectangular function with unit duration, $f_{m}=1/T_{m}$ is the modulation frequency, and $\Delta \Gamma =\Gamma_{\mathrm{ON}}-\Gamma_{\mathrm{OFF}}$ is the difference between the reflection coefficients for the two switch states.

The backscattered field $E_{s}$ can be written as follows:

(4) $${\bar{E}}_{s}\left(t\right)=Re\left[\left({\bar{E}}_{est}+{\bar{E}}_{m}\cdot \sum_{n=-\infty }^{\infty }c_{n}e^{\frac{j2\pi n}{T_{m}}}\right)e^{j2\pi f_{c}t}\right]$$

Therefore, the received signal $x_{R}\left(t\right)$ is the signal backscattered by the tag, attenuated due to propagation and delayed. The delay τ=2r/c depends on the distance between the transmitter and the tag:

(5) $$r=r_{0}+v\cdot t$$

where $r_{0}$ is the initial distance between the radar and the target or tag, and *v* is the relative velocity between both. The effect of the movement adds a frequency shift $f_{D}$ to the frequency components of the received signal due to the Doppler effect, given approximately by the following:

(6) $$f_{D}\approx -\frac{2v}{\lambda }$$

where λ is the wavelength at the center frequency. The sign of the Doppler frequency shift depends on the direction of movement.

The effect of the modulation causes the appearance of the sidebands. The response for the term n=0 is superposed on the structural mode associated with the tag seen as a passive target. Since the amplitude of the Fourier coefficients decreases very fast with the index *n*, in practice, only the first n=±1 can be detected. Depending on the parameters, these sidebands terms can be used to generate ghost responses that can confuse the radar.

**Figure 5:**
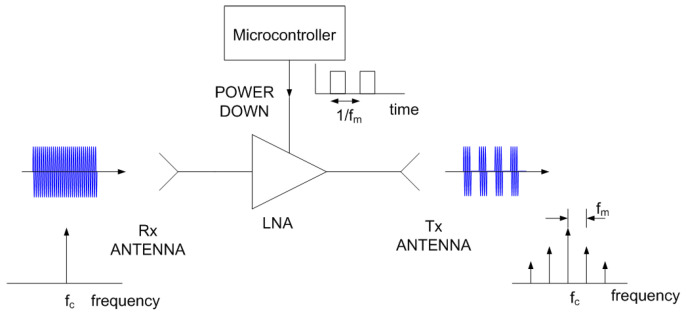
Block diagram of a modulated backscatter based on an amplifier.

Other backscatter topologies can be used to increase the signal level by placing an amplifier between the tag’s receiving and transmitting antenna (see Figure 5). In this case, the modulation can be carried out by means of a switch or, as in this work, by changing the power supply of the amplifier, taking advantage of its isolation in reverse.

The amplitude of this response is proportional to the differential radar cross section given by the following:

(7) $$RCS_{dif}=\frac{\lambda^{2}}{4\pi }G_{Rx}G_{a}G_{Tx}m$$

where $G_{Rx}$ and $G_{Tx}$ are the gains in the receiving and transmitting antennas of the tag, and $G_{a}$ is the gain of the amplifier. *m* is a modulation factor that depends on the Fourier coefficient, and $m=1/\pi^{2}$ for a square wave waveform [50]. This equation suggests that the level of interference could be increased by increasing the gain in the antennas or that of the amplifier. The advantage of using an amplifier in the transponder is obvious compared with passive backscatter methods ($G_{Tx}=G_{Rx}$ and $G_{a}=1$). The excess power required due to the amplifier is not usually a problem in this case, unlike conventional RFID applications.

Due to the propagation between the input and output antenna in the semi-passive transmission backscatter used in this work, the modulated signal includes an additional delay compared to the case of a passive reflection backscatter. This delay must be added to the propagation delay between the tag and the radar (τ). Since the distance between antennas is only a few centimeters, this delay is difficult to resolve due to the limited resolution of the radar. Consequently, it can be considered as a small error in the position of the spoofing target.

**Figure 6:**
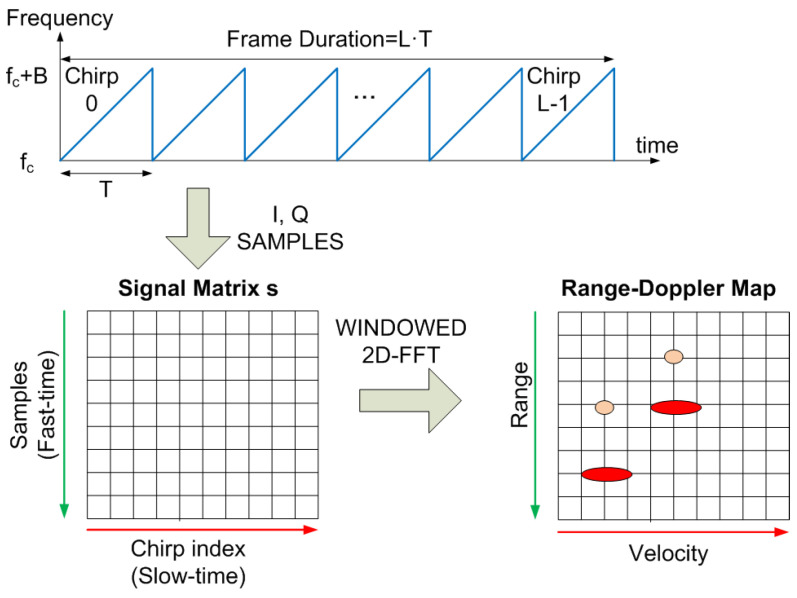
Diagram of the transmitted signal and calculation of the range–Doppler matrix from the I and Q samples received.

FMCW radar uses a range–Doppler (RD) map to simultaneously detect the range and velocity of the targets [56] obtained from the samples of several chirps grouped in a frame (see Figure 6). Each frame is composed of *L* chirps with *N* samples per chirp sampled at a rate of $f_{s}=1/T_{s}$. The complex representations (s[n]=I[n]+jQ[n]) are saved in a matrix *s* so that the columns correspond to the samples for each chirp transmitted.

For a single backscatter case at distance $r_{0}$ and under some typical approximations that ignore second order contributions, the elements of this matrix can be written as [57]:

(8) $$\begin{array}{c} s\left(k,l\right)=A\prod \left(\frac{kT_{s}}{T}\right)\cdot \sum_{n=-\infty }^{\infty }c_{n}e^{-j2\pi \mu \tau_{0}kT_{s}}e^{j2\pi f_{D}lT}e^{j2\pi nf_{m}\left(kT_{s}+lT\right)} \end{array}$$

where k=0...N−1, l=0...L−1, and $\tau_{0}=2r_{0}/c$. If more targets or backscatters are considered, the signal would be the sum of the contributions applying the superposition principle, taking into account the corresponding delay and attenuation.

After that, the RD map is obtained from the windowed two-dimensional Fourier transform of *s*. Hann’s window functions in both range and Doppler are often used to reduce sidelobes in the Fourier transform [58], and zero-padding is used to interpolate the frequency bins when the transform is carried out. The RD map is obtained from the next operation:

(9) $$\mathrm{RD}={\mathrm{FFT}}_{\mathrm{doppler}}\left({\mathrm{FFT}}_{\mathrm{range}}\left(s\cdot w_{\mathrm{range}}\right)\cdot w_{\mathrm{doppler}}\right)$$

where FFT${}_{\mathrm{range}}$ and FFT${}_{\mathrm{doppler}}$ denote the FFT along the range index *k* and the Doppler index *l*, and $w_{\mathrm{range}}$ and $w_{\mathrm{doppler}}$ are the windows used in range and Doppler (e.g., Hann’s window), respectively. The range *R* and velocity *v* depend on the 2D Fourier frequencies, $f_{r}$ and $f_{v}$, respectively, and are obtained from the following expressions:

(10) $$R=\frac{cf_{r}}{2\mu }$$

(11) $$v=-\frac{\lambda f_{v}}{2}$$

**Figure 7:**
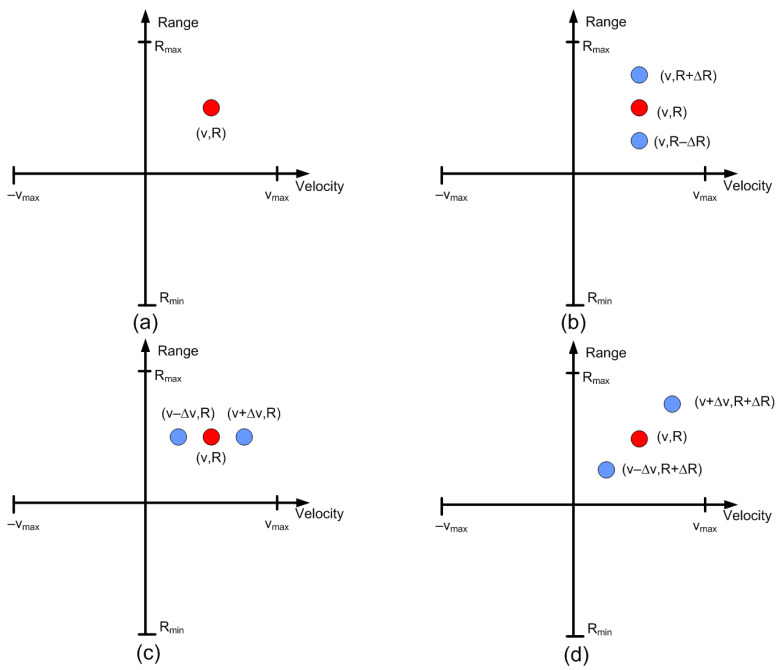
Ghost points introduced for the backscatter: (**a**) unmodulated case, (**b**) high-frequency modulation case with a modulation frequency integer multiple of $f_{sd}/2$, (**c**) low-frequency modulation case, and (**d**) high-frequency modulation case where the modulation frequency is not an integer multiple of $f_{sd}/2$.

The position of targets on the RD map can be detected by analyzing (8) and Figure 7. Of note, unmodulated passive targets are considered for the case of n=0. A peak in the RD map associated with the point closest to the pair of frequencies $f_{r}=2R\mu /c$ and $f_{D}$ is observed. These frequencies correspond to the point with coordinates (R,v) on the RD map (see Figure 7a). The backscatter modulation produces a frequency shift over the beat signal at the output of the mixer. Fast frequency components in the beat signal are interpreted by the radar as changes due to the delay produced by the propagation distance between the radar and the target, whereas smooth phase changes between chirps are associated with Doppler variations caused by the velocity at which the target moves. Three cases can be considered for the modulated backscatter. The first corresponds to a high modulating frequency, in which its value is an integer that is a multiple of half of the sampling frequency in Doppler ($f_{sd}=1/T$). The half of the sampling frequency in Doppler corresponds to the Nyquist frequency in this axis. In this case, sidebands $f_{m}$ apart on the range frequency axis are observed, producing ghost peaks on the RD map with coordinates (R+nΔR,v) (see Figure 7b). The second case occurs when $f_{m}<1/T$ and corresponds to the case of low-modulation frequencies in which the values are of the order of the changes produced in the Doppler frequency. Therefore, due to its low-modulation frequency, the phase of this beat signal does not produce a significant change within each chirp (therefore, the range does not change with respect to an unmodulated tag) but introduces a slow phase variation between the chirps in the frame that can be interpreted by the radar as a Doppler shift when the Fourier transform is performed in Doppler. Consequently, two sidebands $f_{m}$ apart on the Doppler frequency axis appear, producing ghost peaks in the RD map with coordinates (R,v+nΔv) (see Figure 7c). The third case, shown in Figure 7d, is a combination of the cases from Figure 7b,c, where the modulation frequency is high but not an integer that is a multiple of half of the sampling frequency in Doppler. In practice, the first sidebands (n=±1) can only be detected for typical ranges because the harmonics fall below the clutter.

For a certain spacing in the range ΔR and velocity Δv between the ghost points and unmodulated point on the RD map, the backscatter modulation frequency $f_{m}$ is chosen using the following expression:

(12) $$f_{m}=\left[\left(2\mu \cdot \Delta R/c\right)÷\left(f_{sd}/2\right)\right]\cdot \left(f_{sd}/2\right)-2\Delta v/\lambda$$

where ÷ is the integer division. The first term of (12) determines a shift in range. The spoofed target position is rounded to the nearest modulation frequency that does not produce a Doppler shift. Then, a frequency shift is added to produce a shift in the velocity axis.

Therefore, by changing the modulation frequency, the introduction of false targets on the RD map around the physical position of the backscatter is possible. Thus, a backscatter-based tag located in a car or on the road can introduce points that can generate signals that confuse the radar, compromising the safety of the vehicle. The modulation frequency can be easily changed by generating a pulse-width modulation (PWM) at the desired frequency using a low-cost and low-power microcontroller. Different frequencies profiles can be synthesized by varying the PWM signal as a function of time.

## 3. Spoofing Device Design

The spoofing device was based on an updated version of the backscatter tag presented in [59,60]. The GaAs amplifier was replaced by a pair of lower-power-consumption amplifiers, and the patch array was replaced by a lens antenna. The prototype used a silicon germanium MMIC (monolithic microwave integrated circuit) amplifier LNA_24_04 from Silicon Radar GmbH. The typical gain of this amplifier between 24 GHz and 29 GHz is 17 dB, with only 5.6 mA of current consumption at 3.3V. A second cascade amplifier was added to increase the differential radar cross section according to (7). The amplifier has a shutdown power-down control pin that is used to enable/disable the amplifier. With this method, quick, pulsed operation of several megahertz can be achieved, which is sufficient for our application. Therefore, adding an expensive mm wave modulator to the system is not necessary, since modulation of the RCS is carried out by switching the power-down pin of the amplifier. Consequently, the amplitude of the backscattered field can be controlled by a modulation signal connected to the power-control pin, as shown in Figure 5. A low-cost Seeduino XIAO is used to generate the frequency-configurable PWM output. This module integrates an Atmel ARM Cortex-M0 ATSAM21G18A-MU microcontroller operating at a clock frequency of 48 MHz. Of note, other microcontrollers that have PWM outputs and similar performance can also be chosen for this prototype. Generating different ghost targets at distances and velocities according to (12) is possible by choosing the appropriate PWM frequency.

The input and output antennas of the prototype are integrated lens antennas (ILA). The antenna consists of a microstrip patch with a lens to increase the gain [61,62,63]. The material used to manufacture the low-cost lenses using a 3D printer was polylactic acid (PLA). The dielectric data for PLA at millimeter band can be found in [64]. A dielectric constant of 2.55 and a dissipation factor of 0.02 were considered for the PLA material at 24 GHz for the antenna design. The lens was illuminated by a microstrip patch antenna designed on Rogers 4003 substrate with a thickness of 16 mm, a dielectric permittivity of 3.59, and a dissipation factor of 0.003.

**Figure 8:**
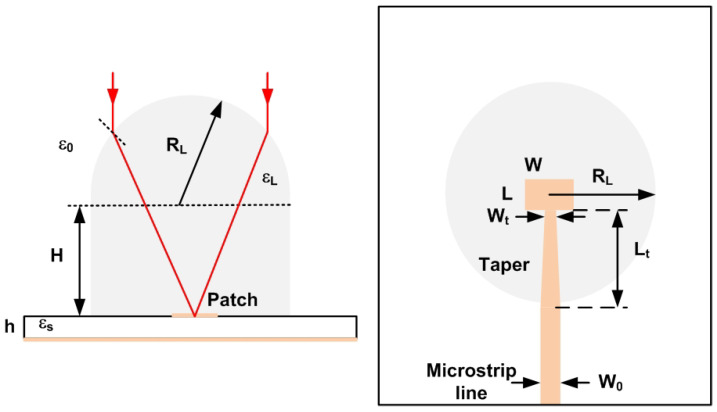
Geometry and dimensions of the antenna.

Figure 8 schematically shows a ray-tracing analysis of the focal length of the extended hemispherical lens. The optimal size of the lens extension of a conventional elliptical lens is given by the following [62]:

(13) $$H_{L}=R\left(\frac{\sqrt{\epsilon_{rL}}+1}{\sqrt{\epsilon_{rL}}-1}-1\right)$$

where $R_{L}$ is the radius of the lens in the vertical plane, *H* is the length of the extension from the substrate, and $\epsilon_{rL}$ is the permittivity of the lens material (PLA). The radius $R_{L}$ was fixed to the wavelength ($\lambda_{0}$) in air at 24 GHz, and *H* is obtained from 13. The patch was designed by taking into account that the PLA structure of the lens lays on it. The width of the patch *W* is assumed to be equal to half of the wavelength when considering an effective permittivity approximately equal to the average between the lens material (PLA) and the substrate $\epsilon_{rs}$:

(14) $$W=\frac{c}{2f_{0}\sqrt{(\epsilon_{rL}+\epsilon_{rs})/2}}$$

Taking this width into account, a better estimate of the effective permittivity $\epsilon_{ref}$ from the effective permittivity of a microstrip line (case W>h) is possible [65]:

(15) $$\epsilon_{ref}=\frac{\epsilon_{rs}+\epsilon_{rL}}{2}+\frac{\epsilon_{rs}-\epsilon_{rL}}{2}\cdot {\left(1+12h/W\right)}^{-1/2}$$

where *h* is the thickness of the substrate. The patch length was designed to be resonant at $f_{0}$ = 24 GHz. Therefore, the effective length must be half of the wavelength calculated from the effective dielectric permittivity. To adjust the resonance frequency to the desired frequency ($f_{0}=$ 24 GHz) and to take into account the fringing effect in the ends of the patch, a length extension (ΔL) is considered [65]:

(16) $$L=\frac{c}{2f_{0}\sqrt{\epsilon_{ref}}}-2\Delta L$$

where the length extension is estimated as [65]:

(17) $$\Delta L=0.412h\frac{\left(\epsilon_{ref}+0.3\right)\cdot \left(W/h+0.264\right)}{\left(\epsilon_{ref}-0.258\right)\cdot \left(W/h+0.8\right)}$$

**Figure 9:**
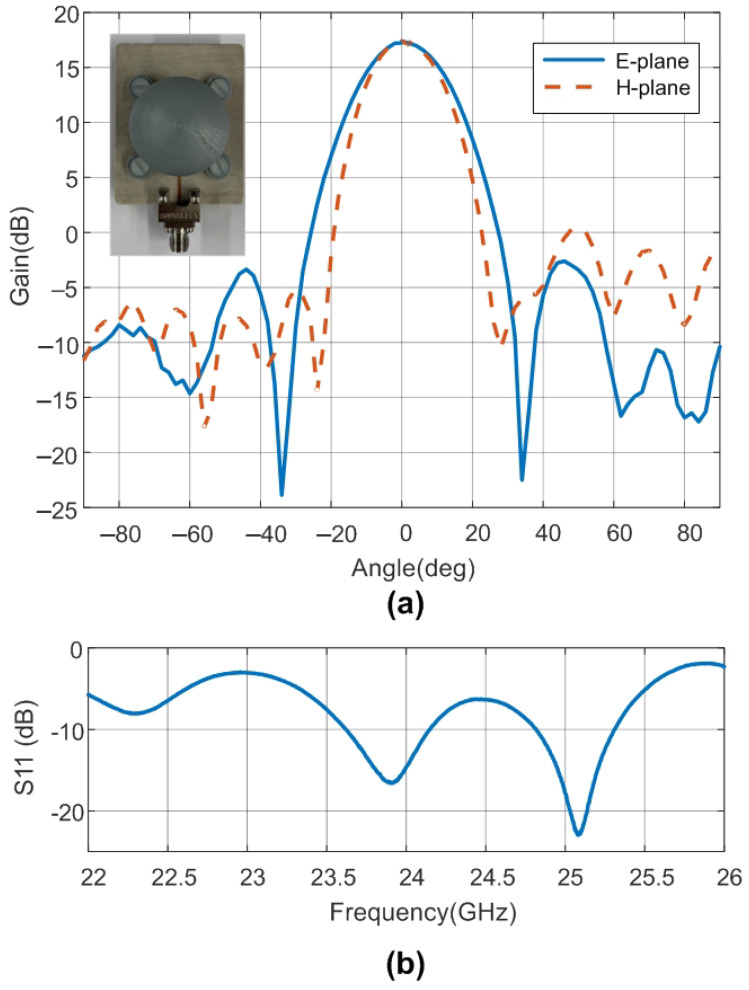
(**a**) Measured gain in the lens antenna (cuts in the E-plane and H-plane). Inset image of the antenna prototype. (**b**) Measured reflection coefficient as a function of the frequency.

The design of the patch antenna, including the effect of the lens material and taper, was carried out with the Momentum software included in the Keysight ADS and with Ansys HFSS. A prototype antenna was manufactured, the sketch of which is presented in Figure 8. The gain and pattern diagram measurements were performed in a semi-anechoic chamber to avoid reflections of the environment, and a calibrated horn antenna was used in the gain calibration. Measurements were performed using a PCB end launch connector (2.92 mm (K)) from Southwest Microwave. The measured gains at 24 GHz in the E-plane and H-plane are shown in Figure 9. The antenna has a maximum gain of 17.5 dB, a sidelobe level better than 18 dB, and a −3 dB beamwidth of about 22 degrees in both planes. Figure 9 also shows the measured reflection coefficient of the prototype (S11) including the end launch connector. The return losses are better than 10 dB in the 24 GHz ISM band. The vector network analyzer (VNA) PNA E8364C from Agilent was used to perform these measurements.

**Figure 10:**
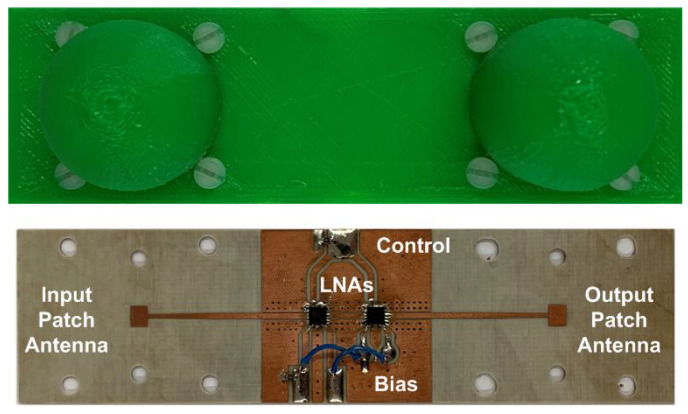
Photo of the prototype.

**Figure 11:**
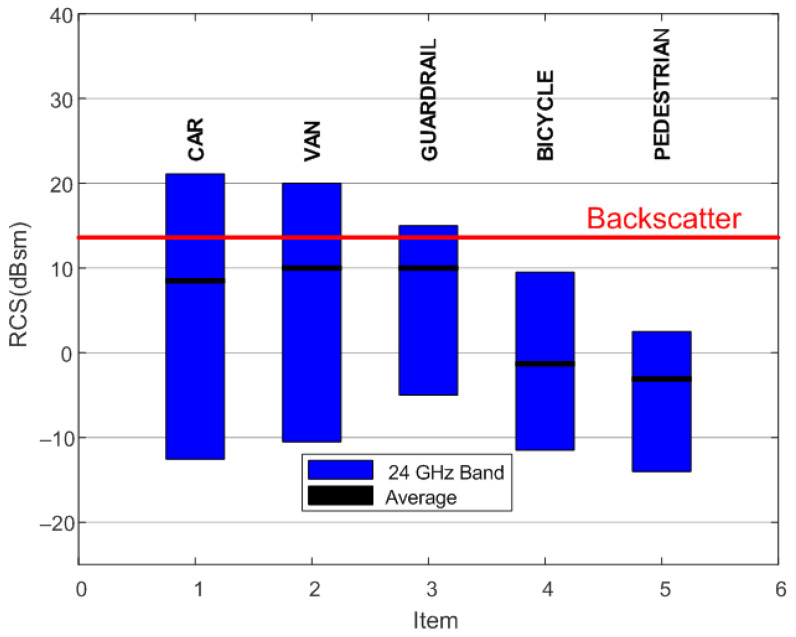
RCS level of different relevant road objects such as a car, a small van, a guardrail, a bicycle, and a pedestrian at 24 GHz band.The peak differential radar cross section for the prototype of modulated backscatter is shown as a red line.

Table 1 summarizes the dimensions of the antenna. Figure 10 shows a photograph of the prototype spoofing device. The dimensions of the device are 120 mm × 37 mm × 24 mm, including the protection box. We proved that the isolation between antennas is greater than the gain in the amplifiers, avoiding oscillation of the device due to the parasitic feedback between the output and input antennas. From 6, the estimated differential radar cross section is 13.6 dBsm. Figure 11 compares the typical values of RCS in the 24 GHz band for some of the objects that can be found on the road, taken from the literature. The RCS value depends on different factors such as size, material, or orientation with respect to the radar. Thus, the average RCS of a pedestrian reported in the literature [66] is about −5 dBsm. This value is significantly lower than those for a car (10 dBsm), a motorcycle (5–8 dBsm) [67], or a guardrail (5–10 dBsm) [68]. Therefore, the designed spoofing device based on modulated backscatter has an RCS level comparable with that of a car and, therefore, can be confused with it.

## 4. Results

### 4.1. Simulated Results

**Figure 12:**
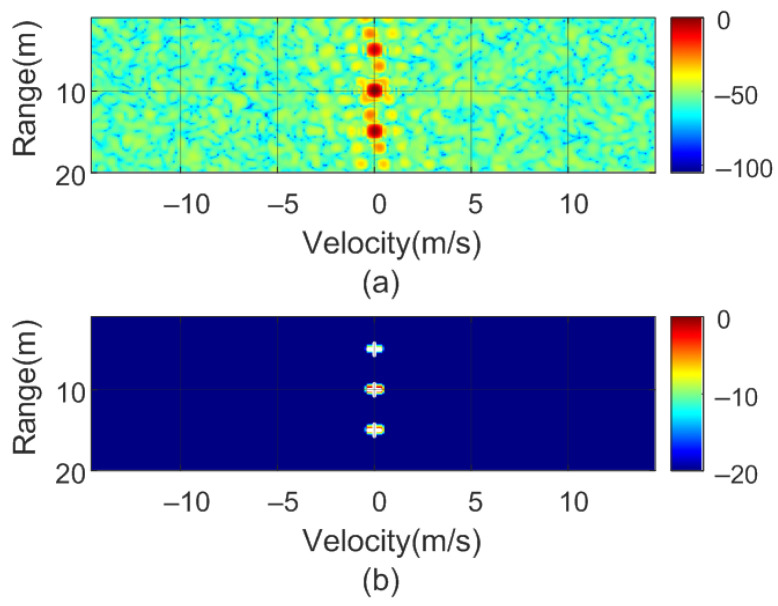
Simulated range–Doppler map (**a**) and after CFAR detector (**b**) for case a in Table 2.

**Figure 13:**
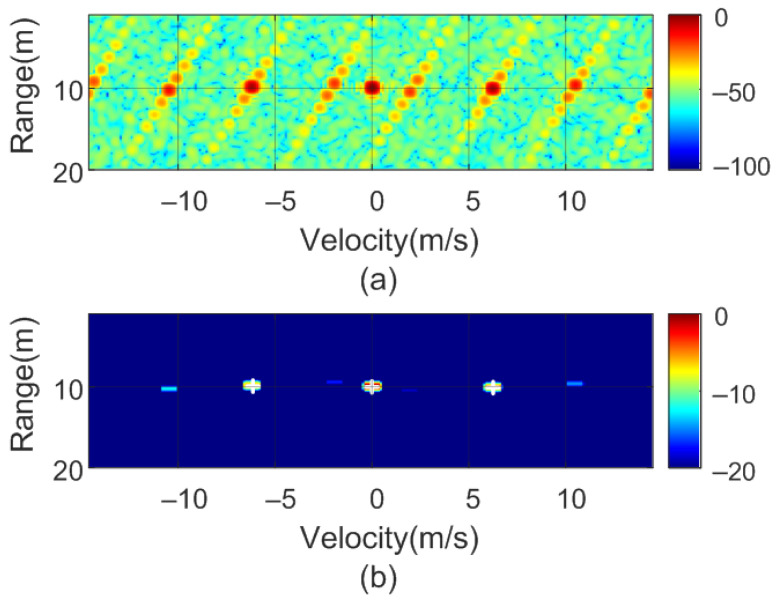
Simulated range–Doppler map (**a**) and after CFAR detector (**b**) for case b in Table 2.

**Figure 14:**
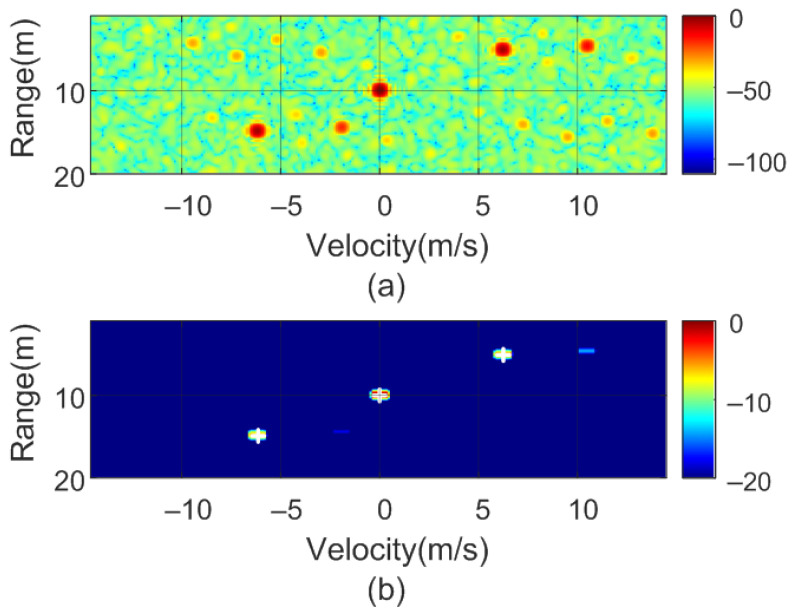
Simulated range–Doppler map (**a**) and after CFAR detector (**b**) for case c in Table 2.

**Figure 15:**
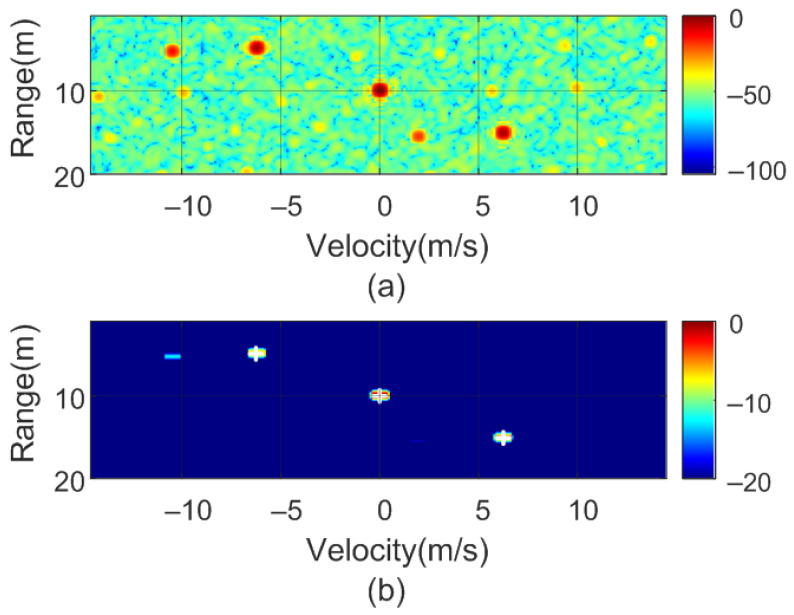
Simulated range–Doppler map (**a**) and after CFAR detector (**b**) for case d in Table 2.

To verify the previous theory, a set of simulations were carried out, in which the presence of cluttering interference from the scene was not considered. The synthetic signals received were simulated from each target using (1)–(6). The data received were sampled at a rate of $f_{s}$, and from (8), the 2D-FFT was performed to obtain the range–Doppler map. In the simulation, parameters similar to those used by the radar during the experiments were chosen. Therefore, a total of 128 frames were taken with 256 points per frame in the next simulations. The radar sweeps from the 24 GHz to 24.3 GHz frequency bands with a sweep time of 250 μs. Figure 12, Figure 13, Figure 14 and Figure 15 show the RD maps obtained for different modulation frequencies corresponding to the cases described in Figure 7. Table 2 lists the frequencies and the values of the offset positions ΔR and the offset velocities Δv. A stationary spoofing device located at 10 m is considered in these examples. The figures show both the raw RD maps and these maps after having applied a CFAR detector. This detector is used to estimate a threshold that eliminates unwanted interference and sidelobes associated with Fourier transforms of the windows. The threshold is obtained from the RD of the adjacent cells. Several methods exist, but in this work, the well-known cell-averaging (CA-CFAR) method with 20 dB above the threshold and 15 averaging cells in addition to two guard cells in both range and velocity was considered. A centroid method was used to determine the position of the main targets. The positions of the detected centroids are shown and marked with a cross.

### 4.2. Experimental Validation

**Figure 16:**
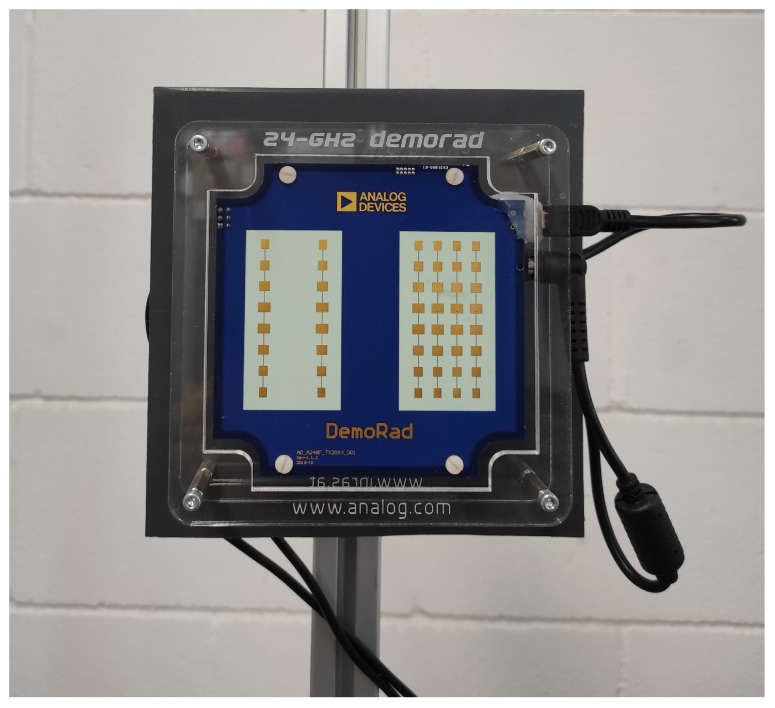
Image of EVAL-DEMORAD kit used in the experiments.

**Figure 17:**
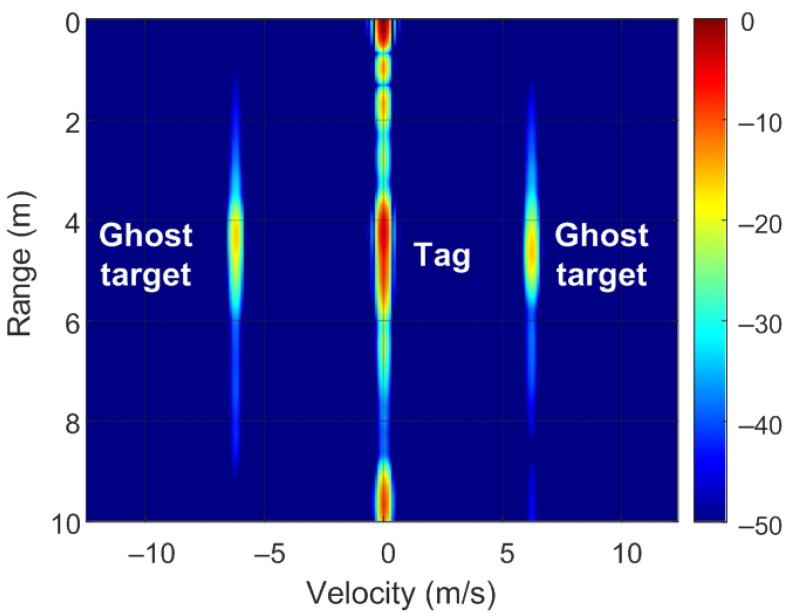
Measured range–Doppler map including two ghost targets generated with a Doppler frequency shift of 1000 Hz keeping the tag static.

**Figure 18:**
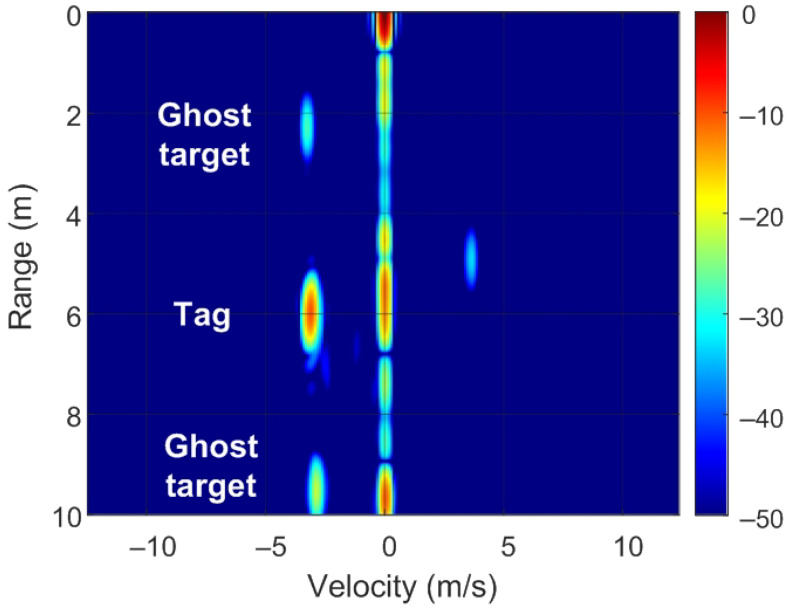
Measured range–Doppler map including two ghost targets at 3.5 m with the tag approaching the radar.

**Figure 19:**
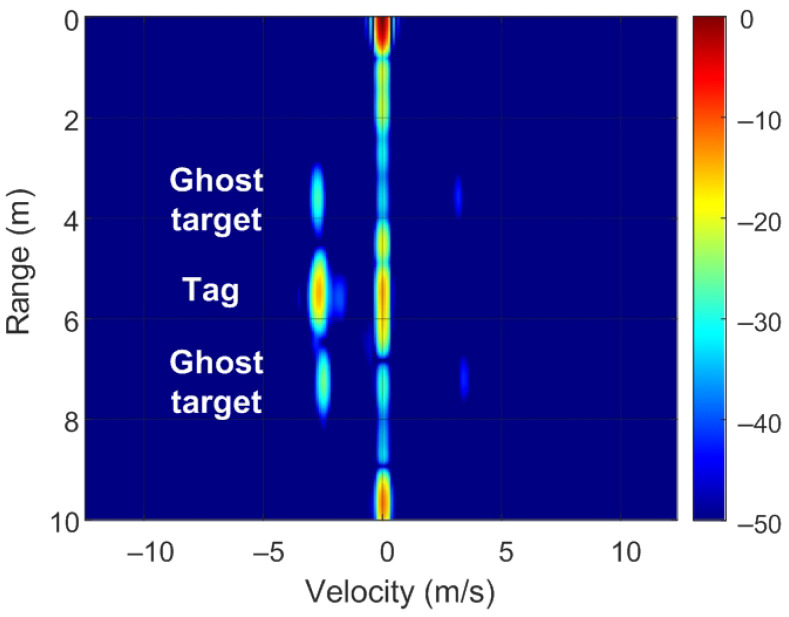
Measured range–Doppler map including two ghost targets at 2.5 m with the tag approaching the radar.

**Figure 20:**
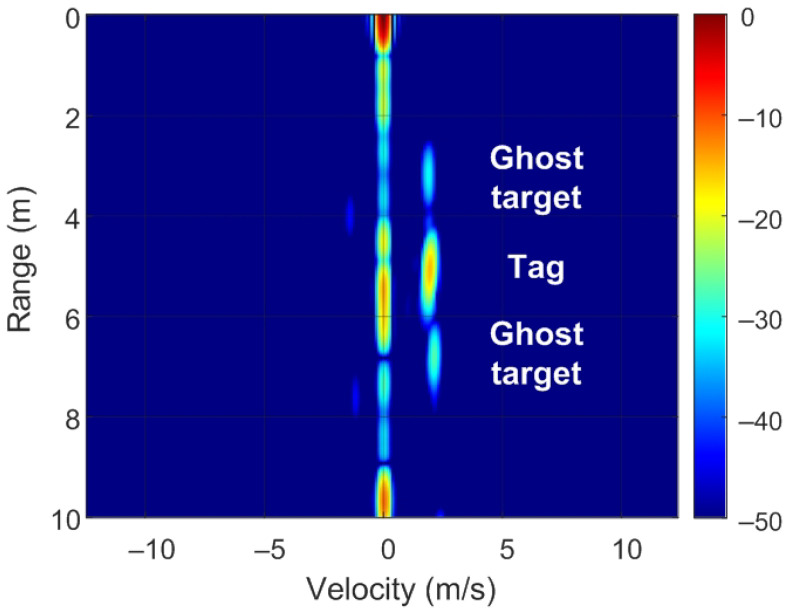
Measured range–Doppler map including two phantom targets at 2.5 m with the tag moving away from the radar.

**Figure 21:**
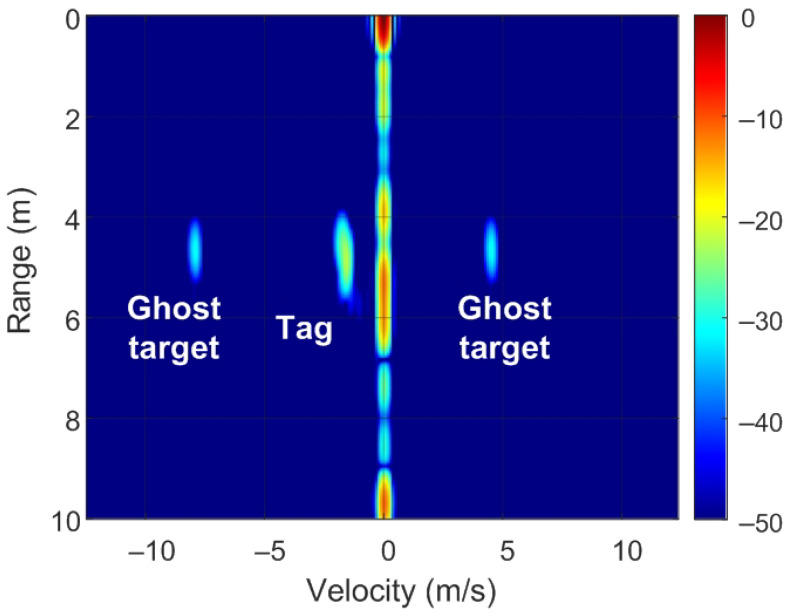
Measured range–Doppler map including two ghost targets generated with a Doppler frequency shift of 1000 Hz with the tag approaching the radar.

For the spoofing validation, a FMCW radar kit (EVAL-DEMORAD) from Analog Devices Inc. (Norwood, MA, USA) was used (see Figure 16). The radar consists of two transmitters and four receiver antennas that are based on the ADF5901 2-channel FMCW transmitter and the ADF5904 4-channel receiver, both from Analog Devices Inc. The radar uses a patch antenna array design with a field of view (FOV) of approximately 120 degrees in azimuth and 15 degrees in elevation. The power transmitted by the radar is 8 dBm. The FMCW radar was configured to sweep within the ISM frequency band (24–24.3 GHz). The sampling frequency was 1.2 MHz, and the sweep time used was 250 μs. The points per frame were 256, and a total of 128 frames were used to obtain the range–Doppler maps. The main parameters are summarized in Table 3. The designed spoofing device and the commercial radar kit were used in the experiment. A test software developed with Python and Matlab based on the communication functions provided with the kit was developed. The experiments were performed in an indoor environment (research laboratory). The following figures depict several cases for different modulation frequencies obtained from (12) for a range ΔR and a velocity Δv. Figure 17 shows the measured RD map after applying the CFAR detector considering a $f_{m}$=1000 Hz. Therefore, according to the theory presented, two ghost targets are observed at the same distance from the tag or spoofing device (ΔR=0) but with a Doppler shift corresponding to δv= 6.25 m/s. A second case is presented in Figure 18. This case shows a spoofing device located at 6 m from the radar. It is also in motion to avoid cluttering interference due to static objects in the laboratory and thus to better appreciate the results. A pair of ghost targets at ΔR=3.5 m without Doppler shift (Δv=0) are generated. The Doppler shift is observed to be due to movement of the tag or spoofing device. The spoofing device is held by a walking person. Figure 19 and Figure 20 show the same case but with the spoofing device moving in both directions (towards and away from the radar, respectively) with ΔR=2.5 m and Δv=0. Figure 21 shows another case where a 6.5 m/s Doppler shift ($f_{m}$=1000 Hz) was introduced into a moving spoofing device (tag).

**Figure 22:**
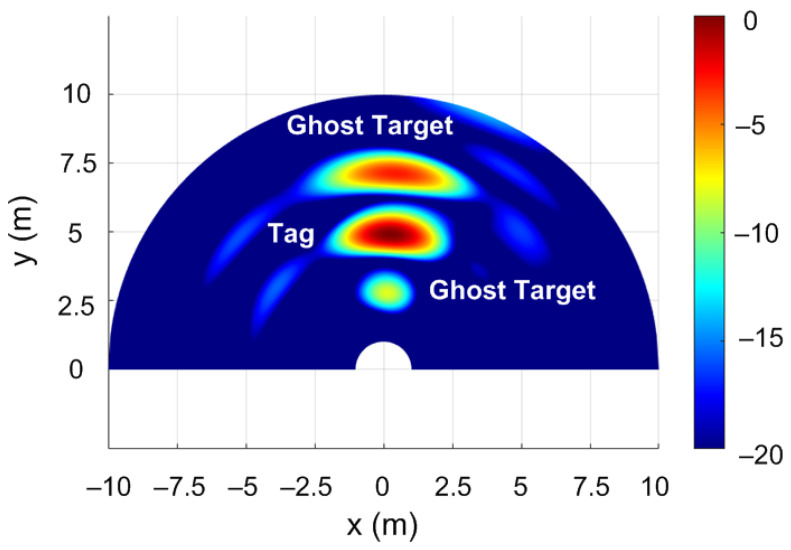
Measured range–angle map including two phantom targets at 2.5 m.

FMCW radars equipped with multiple receive and transmit antennas can estimate the angle of arrival (AoA), showing the targets on range–angle maps. The ghost targets generated by the backscatter tag used as a spoofing device can be observed on range–angle maps. The FFT-based algorithm for AoA detection is one of the most widely used algorithms because of its low complexity and ease of implementation [69]. Angle estimation is performed by processing the received signal at the array composed of multiple antennas. If a Fourier transform is performed in the spatial dimension through the receiving elements (known as angle FFT), distinguishing objects based on their AoA in azimuth will be possible. Since the EVAL-DEMORAD kit has two transmitting antennas and four receiving antennas, the angle resolution is limited; however, the ghost targets can be detected. Figure 22 shows the range–angle map obtained from the FFT method using the EVAL-DEMORAD kit. In this example, a tag or spoofing device is located at 5 m from the radar and two ghost targets are generated by programming the modulation frequency to ΔR=2.5 m. The measurements in the last case have been performed in an outdoor scenario.

## 5. Discussion and Countermeasures

Since spoofing attacks on automotive radars can seriously affect safety and may cause accidents that endanger human lives, providing countermeasures to detect and neutralize such attacks is necessary.

Strategies to combat the attacks are derived from Equation (12). From (12), the ghost points on the range axis are at a distance ΔR from the backscatter position, which depends on the slope of the sweep frequency. Analogously, from (12), the ghost points on the velocity axis are Δv from the backscatter position and are a function of wavelength. The attacker does not know these radar parameters a priori, which may be different from one radar model to another. However, the number of existing automotive radars is not very high, so fining out the vehicle model from the radar is easy. In addition, the attacker can program a microcontroller with different typical configuration parameters corresponding to various standard radar models to periodically generate attacks.

A countermeasure that the radar can implement, which also prevents interference from other radars, is to vary the slope of the frequency sweep, for example, by changing the duration of the chirp signal between frames. The algorithm compares the RD obtained with different frequency slope chirps. This allows the ghost points to appear at different distances or velocities between two consecutive range–Doppler maps. Consequently, identifying them is easy: by comparing the positions of the detected targets between sweeps.

The case in which the spoofing device introduces false targets at the same position (ΔR=0) but with different velocities (see Figure 7c) can be identified by considering that two peaks appear with the same amplitude but velocities equal to 2Δv but with opposite signs, which cannot correspond to a true target. In this case, the position in the RD map is independent of the sweep slope.

Unfortunately, the radar kit used in our experiments does not allow for arbitrary transmission of the sequences to be chosen, so changing the chirp duration within the frame is not possible. To investigate the effect of the chirp, we could only change the duration between frames, shown in the following simulations.

**Figure 23:**
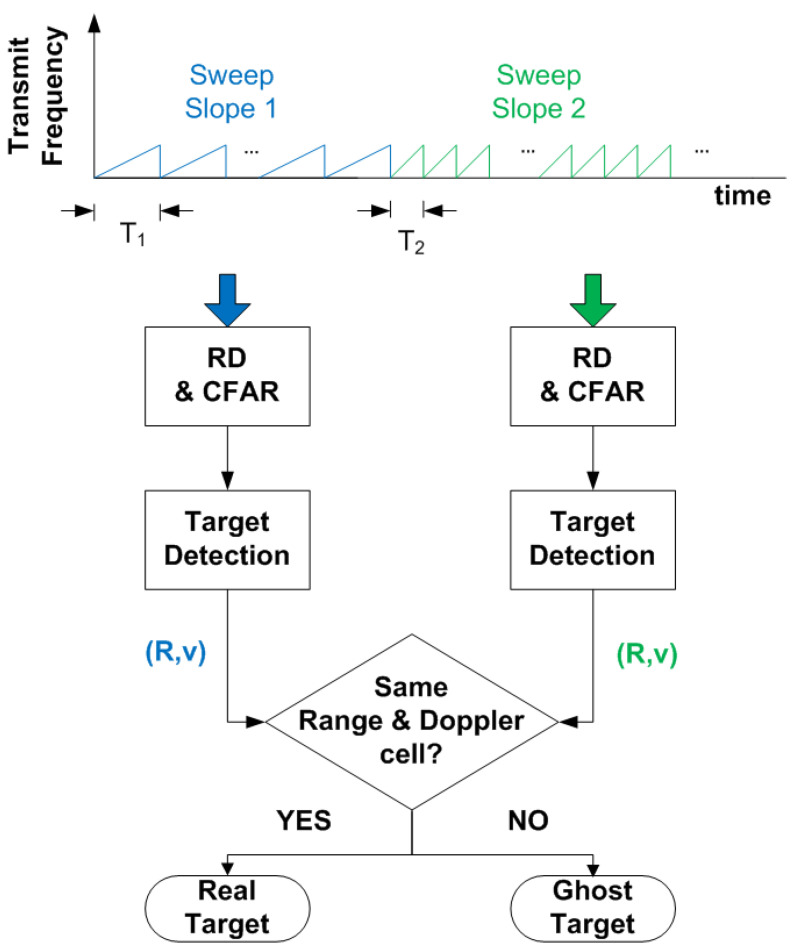
Procedure used to detect a spoofing target using data from two chirps with different sweep slopes.

**Figure 24:**
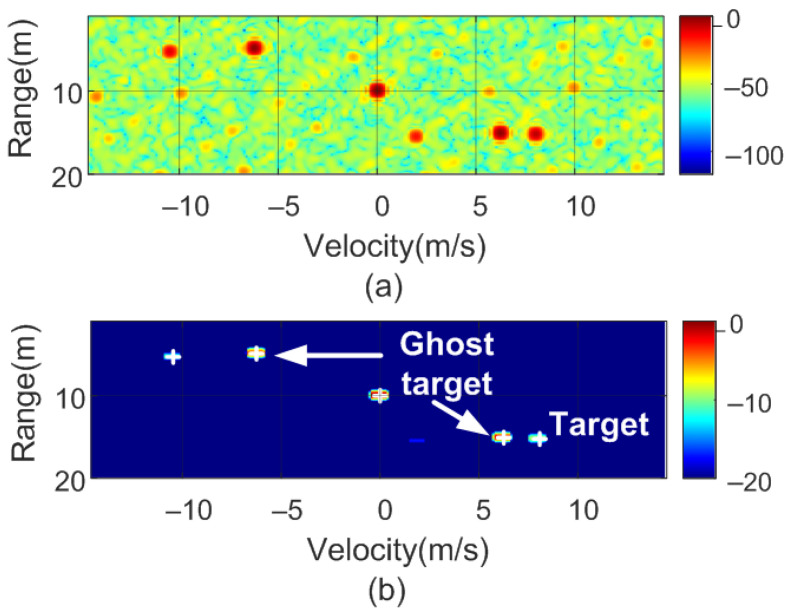
Simulated range–Doppler map (**a**) and after CFAR detector (**b**) with a real target at 15 m and 8 m/s.

**Figure 25:**
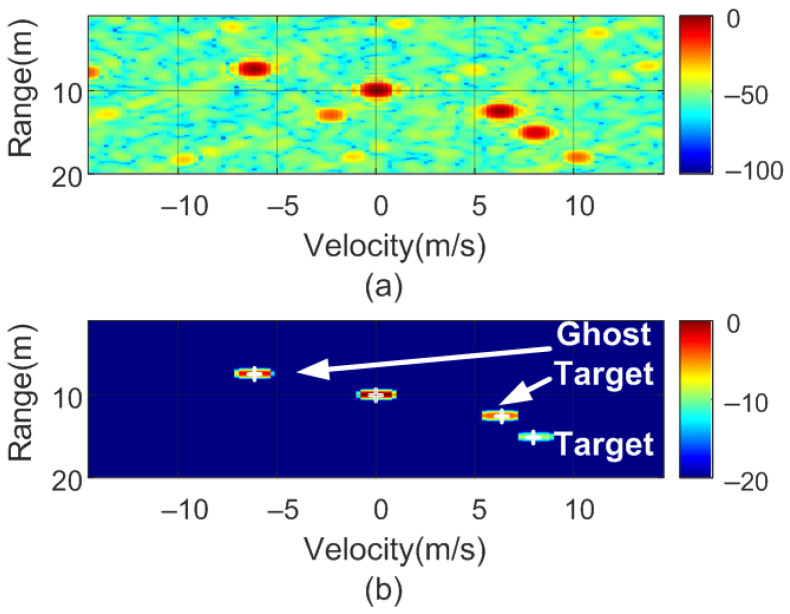
Simulated range–Doppler map (**a**) and after CFAR detector (**b**) with a real target at 15 m and 8 m/s with the slope changed.

Figure 23 shows a schematic diagram of the proposed algorithm for determining if a target is real or spoofed. The objective is to send a set of frames with a specific sweep slope, determining the list of potential targets, and then to send another set of frames with a different sweep slope (or frame duration). An example of the simulations performed is shown in Figure 24 and Figure 25.

In the following example, a real target 15 m away and moving at 8 m/s towards the radar is considered. A stationary spoofing device is located 10 m away. The spoofing device is configured to generate ghost targets with values of ΔR = 5 m and Δv = 6.25 m/s, and a nominal sweep slope μ. The simulated RD is shown in Figure 24. The crosses show the potential targets detected by the algorithm. In subsequent sweeps, the value of the sweep slope is doubled. The corresponding RD is shown in Figure 25. As observed, the real target preserves the same position and velocity, whereas the range of the spoofed targets changes. Consequently, the countermeasure algorithm detects these false targets and discards them. Finally, the use of this algorithm, which is easy to implement, does not prevent normal operation of the radar, the hardware of which remains the same. A similar method used to combat interferences and attackers has been recently proposed in [36] and consists of splitting a chirp into multiple sub-chirps and randomizing every chirp period. Therefore, this random frequency hopping makes it difficult for the attacker to listen (e.g., with an spectrum analyzer or receiver) to the victim’s radar signal to learn the main radar parameters (such as frequency range and slope). Although a backscattering-based method presents great flexibility in the design of the targets, due to the presence of the sidebands (since the modulation is not a single sideband), the targets appear in pairs, and a Doppler coupling can be seen, as shown in Figure 7. Therefore, in the car-follower scenario shown in Figure 2, one target is closer to the radar and the other is behind the attacker. The victim’s radar considers the closest target to be less than a safe distance away. Consequently, the radar alerts a user to the threat of a collision. To avoid the pairs of peaks, more complex single-sideband (SSB) modulators must be implemented. In the case of backscatter in a reflection, they can be implemented using a hybrid coupler loaded with switching devices (e.g., PIN diodes) or using IQ mixers in the case with a transponder in transmission.

## 6. Conclusions and Future Work

In this work, a simple low-cost spoofing device operating at 24 GHz based on modulated backscatter was proposed. Our goal was to generate false targets, thus fooling vehicles that incorporate a mm wave FMCW radar. In contrast with other works in the literature that use SDRs or other radars to generate spoofing signals, we proposed a simple device based on a modulated semi-passive transponder or tag. We demonstrated that, by changing the modulation of this transponder, the generation of ghost targets to confuse the radar at different distances and velocities is possible. The transponder consists of two patch antennas with dielectric lenses to achieve a high gain. Two low-noise amplifiers are cascade-connected between the two antennas. Modulation is achieved by switching the gain of the amplifiers. We showed that the combination of high gains of the antennas with those of the amplifiers allows us to achieve a high differential RCS, comparable with that of a car at 24 GHz. The victim radar detects a beat frequency change associated with the backscatter modulation frequency. This frequency shift results in a ghost target, and the modulation frequency can be set to generate spoofing targets at different distances and velocities. Several simulations have been carried out, and different spoofing measures have been validated using the designed tag and the FMCW radar kit (EVAL-DEMORAD) from Analog Devices. In both cases, RD maps have been generated. The results obtained agree with those expected from theory, both for the simulations and for the measurements after applying the CA-CFAR detection algorithm.

Countermeasures mitigating the effects of potential spoofing using these devices based on random variation in the radar sweep parameters, such as the sweep slope or the duration of the chirps, were proposed.

In addition, the low-cost device presented allows for the generation of artificial targets that can be used to calibrate radars in assembly lines, in technical vehicle-inspection centers, and indoors or to avoid clutter interference as an alternative to expensive commercial instrumentation-based systems.

## Figures and Tables

**Table 1:** Dimensions of the antenna.

Dimension	Value (mm)
$R_{L}$	13.95
H	13.4
L	2.9
W	3.45
$W_{t}$	0.5
$L_{t}$	12.5

**Table 2:** Simulated cases.

Case	Modulation Frequency (Hz)	ΔR (m)	Δv (m/s)
a	46,875	5	0
b	1000	0	6.25
c	46,875 − 1000	5	6.25
d	46,875 + 1000	5	−6.25

**Table 3:** Configuration of the FMCW radar.

Parameter	Value
Start Frequency	24 GHz
Sweep bandwidth	300 MHz
Sweep slope	300/250 MHz/μs
Sweep time	250 μs
Sampling frequency	1.2 Mbps
Number of samples per chirp	256
Number of chirps per frame	128
Transmit antennas	2
Receive antennas	4

## Data Availability

The data presented in this study are available from the corresponding author upon request.
